# The significance of a dementia diagnosis from the perspective of the family caregivers: a qualitative study

**DOI:** 10.1186/s12913-025-12657-1

**Published:** 2025-04-02

**Authors:** Inger Molvik, Grete Kjelvik, Geir Selbæk, Anne Marie Mork Rokstad

**Affiliations:** 1 The Norwegian National Centre for Ageing and Health, Vestfold Hospital Trust, Postboks 2136, Tønsberg, 3103 Norway; 2 Institute of Clinical Medicine, University of Oslo, Oslo, Norway; 3 Department of Geriatric Medicine, Oslo University Hospital, Oslo, Norway; 4 Faculty of Health Sciences and Social Care, Molde University College, Molde, Norway

**Keywords:** Dementia, Cognitive impairment, Next of kin, Experience, Older adults, Timely diagnosis

## Abstract

**Background:**

With the anticipated increase in dementia prevalence over the coming decade, understanding the experience of receiving a dementia diagnosis for people living with cognitive impairment remains limited. This study aims to explore the implications of a family member with cognitive impairment receiving a dementia diagnosis or not from the perspective of their next of kin.

**Methods:**

A qualitative descriptive design was applied using individual interviews for data collection. Participants were recruited based on the cognitive function level of their family members, which was compatible with dementia as assessed with the Montreal Cognitive Assessment Scale (MoCA). The sample consisted of eight participants, comprising family members of five individuals with confirmed dementia diagnoses and three undiagnosed. The analysis was performed using four steps of systematic text condensation to discern codes, categories, and the overarching theme.

**Results:**

Three main categories were created: (1) Impact of observed cognitive decline, (2) Impact of diagnosis on service engagement, and (3) Support and follow-up for family caregivers. The findings show that next of kin who have received a dementia diagnosis for their family members are more proactive in seeking help and services, are better informed about available resources, and are more concerned about future challenges. On the other hand, next of kin to family members without a diagnosis are more inclined to handle the situation on their own, have less access to information and services, and generally express less concern about future problems.

**Conclusion:**

The study reveals the benefits of receiving a timely dementia diagnosis in shaping more effective support systems and policies. This ensures that the next of kin and the person with cognitive impairment can navigate the complexities of dementia with greater confidence and preparedness, thereby enhancing their quality of life.

## Introduction

Despite the expected increase in dementia prevalence over the coming decades [1–4], it is widely acknowledged that dementia remains underdiagnosed, with diagnosis often occurring years after the initial onset of symptoms [5]. This underdiagnosis impedes individuals from understanding their condition and accessing pertinent information and support. According to the Norwegian national guidelines on dementia and the Dementia Plan 2025, an accurate diagnosis is crucial for providing personalized information and support to patients and their families [6, 7]. When cognitive impairments begin to interfere with daily life, obtaining a diagnosis becomes essential for securing appropriate support and follow-up care. A diagnosis can not only enhance access to resources and support but also significantly improve quality of life and support in future planning [8]. However, the emotional and psychological burden of such a diagnosis can be overwhelming, affecting not only the individual but also their family and caregivers, leading to negative outcomes such as anxiety, posttraumatic stress [9–11], and even suicidal thoughts [12].

Receiving a formal diagnosis can also have positive psychological effects. For some people with dementia, receiving the diagnosis provides a sense of relief by validating their experiences and symptoms, allowing them to understand and make sense of their cognitive changes [9, 10]. Studies also show that caregivers feel relieved when a formal dementia diagnosis is made [11]. Many family caregivers find comfort and clarity in the dementia diagnosis, which leads to a deeper understanding and increased patience in their interactions with the person who has dementia [12]. Family caregivers play a critical role in the lives of individuals with dementia, often providing most of the care and support [10, 11]. Their experiences are shaped by the presence or absence of a formal diagnosis [12, 13]. Although most studies have relied on caregiver reports rather than the direct experiences of people with dementia, research exploring the experiences of family caregivers of those diagnosed with dementia versus those living with undiagnosed cognitive impairment remains limited [13]. Understanding these subjective experiences and the impact of a dementia diagnosis is essential for comprehending the role of family caregivers and their need for support and information.

In the context of dementia, a timely diagnosis is defined as providing an accurate diagnosis at a stage in the disease process when it can be most beneficial to individuals with dementia and their families [14–16]. While early diagnosis facilitates access to resources and support, a timely diagnosis is thought to better match the readiness and ability of individuals and their families to benefit from this information [17, 18].

Early diagnosis provides crucial time for adjustment and earlier access to guidance, financial support, and both pharmacological and nonpharmacological treatments [19]. This understanding holds significant relevance not only for individuals diagnosed with dementia but also for their families and healthcare providers. Highlighting the potential benefits of a timely diagnosis involves navigating the delicate balance between providing necessary information and resources at a stage when individuals and their families are ready and able to process it and ensuring that interventions are not delayed to the detriment of the person’s health and wellbeing.

This study aims to explore the implications of a family member with cognitive impairment receiving a dementia diagnosis or not from the perspective of their next of kin. Family members with cognitive impairment may either be diagnosed with dementia or may not have undergone assessment and diagnosis. The focus is to compare the experiences and challenges faced by next of kin in both scenarios, exploring the implications of a formal dementia diagnosis.

## Materials and methods

A qualitative descriptive design [20] was used utilizing individual semi structured interviews [21] to explore the experiences of family caregivers living with or close to persons with cognitive impairment with or without a dementia diagnosis.

### Participants and recruitment

Participants were recruited from the Trøndelag Health Study (HUNT) [22], a longitudinal population health study in Norway that began in 1984 (HUNT1) with new waves every 10 years. In its fourth wave (HUNT4, 2017-19), a sub-study (HUNT4 70+) was conducted, targeting participants aged 70 years and older. A total of 9,956 participants took part in HUNT4 70+. All surviving participants were invited to join a 4-year follow-up study, Ageing in Trøndelag (HUNT AiT (*n* = 5,729)). Data from the cognitive tests in the follow-up study, HUNT AiT, served as the basis for recruiting family caregiver participants. These caregivers were identified through individuals in the HUNT AiT study who met the criteria for cognitive impairment or dementia according to the Montreal Cognitive Assessment (MoCA). Participants were recruited from four municipalities in the Trøndelag region based on their next of kin’s participation in the data collection for the HUNT AiT study. The staff at the field stations in the HUNT AiT study distributed information about this study and an invitation to participate in individual interviews to the participants who met the inclusion criteria of cognitive impairment according to age-adjusted scores falling below the following specified thresholds: 70–79 years < 22; 80–89 years < 21; 90 + years < 20 on the MoCA [23]. During the interviews, they were asked to identify a family caregiver whom our research group could contact for an interview about being the next of kin to a person with cognitive impairment. If they agreed, the next of kin was contacted and invited to take part in an individual interview. The perspectives of people living with cognitive impairment on the impact of getting a dementia diagnosis have previously been presented [24].

In total, 14 participants living with cognitive impairment agreed that next of kin could be contacted. We reached 11 next of kin, of whom eight wished to participate in the study. Among them, five participants were next of kin to persons with an established diagnosis of dementia, whereas the remaining three were next of kin to people not yet diagnosed. Participants supporting family members without a formal dementia diagnosis were not informed of the MoCA scores or their implications. This decision was made to adhere to ethical standards, ensuring that any health assessments and potential diagnoses were communicated only by qualified healthcare professionals to prevent undue stress or misunderstanding.

The sample included three men, of whom two sons did not live with the person with cognitive impairment or dementia, and one male spouse living together with a person with dementia. Additionally, five women, all of whom were spouses living with a person with cognitive impairment or dementia participated. The participants’ characteristics are presented in Table 1 (next of kin to a person diagnosed with dementia) and Table 2 (next of kin to a person not diagnosed with dementia).

**Table 1:** Family caregiver to person diagnosed with dementia

Name	Relation	Gender	Living situation	MoCA-score of the family member with cognitive impairment	Age of the family member with cognitive impairment
Martin	Spouse	M	Living together	10	76
Marvin	Son	M	Not living together	11	81
Molly	Spouse	W	Living together	18	83
Mia	Spouse	W	Living together	8	84
Maria	Spouse	W	Living together	12	80

MoCA: Range: 8–18, mean = 12

Age: Range: 76–84, mean = 81

**Table 2:** Family caregiver to person not diagnosed with dementia

Name	Relation	Gender	Living situation	MoCA score of family member with cognitive impairment	Age of the family member with cognitive impairment
Eric	Son	M	Not living together	20	75
Emma	Spouse	W	Living together	21	76
Eva	Spouse	W	Living together	17	79

MoCA: Range: 17–21, mean = 19

Age: Range: 75–79, mean = 77

### Preunderstanding

The first author (IM) possesses a master’s degree in gerontology and has substantial experience in nursing homes, working with individuals with dementia and their caregivers. The coauthors, GK, GS, and AMMR, bring their expertise from medical research, health, and social sciences. AMMR’s qualifications include registered nursing, and GS specializes in psychiatry. All team members have considerable experience in dementia-related research.

### Interviews

The interviews, conducted by IM, followed a semi structured format with a detailed interview guide [21]. The questions, as outlined in Table 3, were designed to explore the experiences of family caregivers of individuals with cognitive impairment or dementia, specifically in terms of observed alterations in cognition, functionality, and quality of life from the caregivers’ perspective, as well as their personal experiences related to the processes and implications of receiving a dementia diagnosis—or the absence of—a formal dementia diagnosis for the individuals in their care.

**Table 3:** Excerpt from the interview guide

Question
Has your next of kin sought help for problems with memory or orientation – if so, how?
What needs do you perceive your next of kin has in daily life?
What needs do you have as a next of kin (information, guidance, respite, practical help)? • What help and support do you receive to meet these needs (from municipal services, volunteer services, help from others)? • How do you feel your needs as a next of kin are being met?

The interviews were conducted from November 2021 to August 2022 at various locations. One interview was conducted onsite at the HUNT AiT field station, another took place in the participant’s home, and the remaining interviews were conducted by telephone. Before posing any questions, the purpose of the study was re-emphasized to the participants, who were also reminded of their prior consent to participate. Each interview lasted approximately 20–45 min, was audio-recorded, and subsequently transcribed verbatim for analysis.

### Analysis

The analysis of the transcribed interviews was guided by Malterud’s systematic text condensation method [25]. Systematic text condensation provides a structured, rigorous approach, ensuring reflexivity and ease of use. It also enhances credibility through collaborative data interpretation and is outlined in four distinct steps: (1) Reading the material: Thoroughly reading the data, such as interview transcripts, to understand and identify key themes. (2) Identifying meaning units: Break the data into smaller, meaningful units (sentences, paragraphs, or statements) relevant to the research questions. (3) Condensing the units: Summarize these units into shorter, precise statements that retain the original meaning. (4) Synthesizing and systematizing meanings: Organize the condensed units into categories or themes and identify patterns and connections to draw out the main findings and insights (see Table 4 for examples from the analytical process).

**Table 4:** Examples from the analytical process

Data extract	Subcategories	Categories
*We were on holiday*,* and then he started getting confused about where we were staying. How to find the way. And that’s when I started to wonder. However*,* that was 6–7 years ago.*	Recognition of symptoms	Impact of observed cognitive decline
*I know that day programs are available. Respite care is important when needed.*	Information, knowledge and utilization of services	Impact of diagnosis on service engagement
*I have what I need. We are doing well. I am doing well.*	Impacts on caregivers’ quality of life	Support and follow-up for family caregivers

## Results

Three main categories were created from the interviews: (1) Impact of observed cognitive decline, 2) Impact of diagnosis on service engagement, (3) Support and follow-up for family caregivers, as shown in Table 5.

**Table 5:** The significance of a dementia diagnosis from the perspective of the family caregivers

Categories	Impact of observed cognitive decline	Impact of diagnosis on service engagement	Support and follow-up for family caregivers
*Subcategories*	*Recognition of symptoms*	*Information*,* knowledge and utilization of services*	*Support*
	*Initiation of cognitive assessment*	*Expectations and hesitations*	*Impacts on caregivers’ quality of life*
	*Caregivers’ response to the diagnosis*		

### Impact of observed cognitive decline

The category “Impact of observed cognitive decline” is divided into three subcategories: (1) Recognition of symptoms, (2) Initiation of cognitive assessment and (3) Caregivers’ response to the diagnosis.

#### Recognition of symptoms

The catalyst for pursuing a formal cognitive assessment was either witnessing a specific incident or a gradual recognition of symptoms over time that underscored a decline in cognitive ability. This could be changed abilities to keep orientated or decreased functional ability in daily activities. Mia said about her husband: *“He can no longer keep up with the news very well.”* Maria was scared when her husband, who used to know the forest like his own pocket, suddenly did not recognize where he was:

I first noticed it when he didn’t recognize places in the forest and on the mountain. That’s what scared me last year. Last fall. Because when he started asking: “Do you know where the road is?” He’s not allowed to go into the forest alone anymore.

Disorientation and confusion could arise unexpectedly and without an explainable reason. Molly was alerted when her husband was confused on holiday: *“We were on holiday*,* and then he started getting confused about where we were staying. How to find the way. And that’s when I started to wonder. However*,* that was 6–7 years ago.”*

Changes in communication, such as difficulties finding words, were reported in the person with dementia. Furthermore, symptoms such as anger, memory problems and isolation were described. However, Martin did not recognize any symptoms before the diagnosis and expressed that both he and his wife were surprised by the diagnosis process: *“We were not prepared for the diagnosis.”*

Additionally, all caregivers of family members without a dementia diagnosis reported an observed decline in cognition such as memory loss. However, not all of them considered this to be a problem. Eva observed that her husband had trouble with his memory: *“He does not remember everything. He’s a bit forgetful*,*”* but did not consider it a problem: *“He handles things himself and sorts them out. No problem. He’s quite capable.”*

#### Initiation of cognitive assessment

Among the caregivers of family members diagnosed with dementia, three reported that they were typically the first to notice subtle cognitive changes and were instrumental in seeking evaluations from healthcare professionals. Maria stated: *“I was the one who took the initiative to go to the doctor because I noticed that something was different”*.

Other participants did not take direct initiative in assessing the cognitive impairment they observed, but were more kindly encouraging and supportive for the person to seek assessment like one son: *“She has brought it up with the doctor herself*,* but we may have hinted that she should talk to the doctor”* (Marvin).

Some of the participants mentioned that the dementia diagnosis was completely unexpected for them as they did not notice any differences in their family member. They became aware of the changes in cognitive functioning through observations made by others. Mia stated: *“It was our daughter*,* who is a nurse*,* who thought he should see a doctor to have his head checked.”*

Among the caregivers of family members without a dementia diagnosis, none had been assessed for dementia, but Emma said, *“Perhaps we should investigate that.”* Other participants had observed the poor memory of their family member but did not consider it a problem or a need to seek help. Eric was worried about his father and believed that the doctor had misled him. However, his concern was related to his father’s diabetes, which he thought was responsible for the changes that had occurred: *“The doctor has misled my father. Since he got diabetes*,* he has good periods*,* but at times*,* he doesn’t remember things we think he should remember.”*

#### Caregivers’ response to the diagnosis

Caregivers of family members with a dementia diagnosis expressed a range of emotions and experiences regarding the diagnostic process. Martin, who was not prepared and surprised about the diagnosis, said: *“It wasn’t enjoyable to get the diagnosis.”* Other participants thought it was valuable to obtain an assessment even though the message was worrying, and some were anxious or sad about the diagnosis. Mia stated: *“I think it was sad that he got the diagnosis.”* She also worried about her husband not being capable of understanding the full consequences of the diagnosis as he was most concerned about losing his driving licence: *“I’m not entirely sure if he understood it. He almost felt that losing his driver’s licence was more serious than being sick because he did not feel sick.”*

After being diagnosed with dementia, the individuals had received medication to treat or postpone the cognitive impairment. Marvin stated that his mother had improved in speaking after she started dementia medication and that otherwise, there were few changes: *“She carries on as she always has; washing and ironing clothes and cooking.”* He also reported not being informed about the dementia diagnosis, even though his mother received services from the memory team, who provided her dementia medication: *“She receives dementia patches that are changed once a day*,* but I have not heard if she has received a dementia diagnosis.”* This lack of information was also described by another participant. Maria’s husband was prescribed medication for dementia, but neither she nor her husband perceived a clear diagnosis. However, Maria did not question the fact of a dementia diagnosis: *“It’s not such a surprising message to receive when you’re over 80.”*

### Impact of diagnosis on service engagement

The category “Impact of diagnosis on service engagement” is divided into two subcategories: (1) Information, knowledge and utilization of services and (2) Expectations and hesitations.

#### Information, knowledge and utilization of services

Although all participants were supporting family members with cognitive impairment, there was a significant difference in knowledge about various service options among the participants. Next of kin of family members with a dementia diagnosis were generally well informed about service options: *“I know that day programs are available. Respite care is important when needed”* (Maria). Most of them were in regular contact with the case manager or memory team: *“We have visits from the memory team every six months”* (Mia). The participants emphasized the importance of diagnosis as it made a difference in getting in contact with the health and social care services in the municipality.

In contrast, caregivers of family members without a dementia diagnosis faced more uncertainties about the diagnosis process and available services. Eric was unsure if his father discussed memory loss with his GP: “*I don’t know if he has brought up these memory problems with his doctor. I don’t think he has been evaluated.”* One wife was in doubt about seeking help for her family member as she felt content. Another stated that she preferred to manage the situation on her own: *“We manage on our own. I see what needs to be done and lend a hand.”* All three stated that they did not perceive major challenges in their situation. One explained the cognitive impairment as a natural part of getting old. The other two stated that they did not need any help from anyone other than their family. *“We don’t need any help right now. I cook*,* clean*,* and keep the house in order”* (Emma). *“We live in the countryside*,* but we have people around us. We grow potatoes. We live in a big house by ourselves. It’s not a problem”* (Eva).

Service utilization varied among caregivers for those with a dementia diagnosis. Mia’s husband attended a day care centre weekly, whereas Martin received assistance with cleaning and medications. Marvins’ mother also received help with her dementia medications. Molly’s family managed without municipal services as their daughter-in-law organized medications. Maria did not use services but knew help was available: *“We don’t have any services*,* but I know I can contact the municipality and the family doctor if it gets worse.”*

Caregivers of family members without a dementia diagnosis relied mainly on informal help: *“We have children and grandchildren who can help out”* (Emma). Even though those with a dementia diagnosis were aware of respite care they often didn’t see the need or they hesitated to seek more help as they relied on vigilance from the memory team or GP for any changes to increase the need for additional services: *“I hope the memory team or the GP informs us if they notice any changes”* (Marvin).

For one of the caregivers without a dementia diagnosis, there was a desire for better communication with healthcare providers: *“I would like to talk to his general practitioner”* (Eric). The two wives were more confident in managing without external services: *“We do not have any services right now. We manage on our own. We shop for ourselves. We do everything ourselves.”*

#### Expectations and hesitations

Among the caregivers to family members who had not yet received a diagnosis, the son hesitated. Although he had not thought about dementia as a plausible explanation for his father’s problems, he would like to talk to his father’s GP: *“I don’t think he has dementia. I would like to talk to his general practitioner. I don’t know if I can trust that he is as healthy as he says.”* Eva reflected on her husband’s condition and found it difficult to explain:

He often responds with ‘I don’t know anything about that’, but he doesn’t have trouble finding his way home. If it becomes an issue, we will contact the doctor. It’s not easy for me to confirm exactly how things are.

Caregivers to family members with a dementia diagnosis expressed concern about the progression of the disease: *“I hope he doesn’t undergo a personality change”* (Maria). Marvin, who lives close to his mother, has made some adaptations to her home that he hopes will enable her to stay there longer. Martin reflected upon the consequences of the dementia diagnosis: *“We know which way it’s going. Hopefully*,* it will progress slowly.”* Mia expressed her feeling of a big responsibility for both her and her husband’s wellbeing as she had to be the motivator for her husband to take part in daily activities like going for a walk. Molly is quite content and trusts that they will receive the help they need when they need it: *“We do as we please and get help if we need it.”*

### Support and follow-up for family caregivers

The category “Support and follow-up for family caregivers” is divided into two subcategories: (1) Support and (2) Impacts on Caregivers’ Quality of Life.

#### Support

Caregivers of family members with dementia felt well informed about the disease and the services offered. Almost all caregivers of family members with a dementia diagnosis had attended the caregiver education and found it useful: *“I benefited from attending school for family caregivers. It was helpful to see that some have it worse than you”* (Mia). Molly also attended the same kind of school together with her children. Maria was aware of this available support and would consider attending later on: *“I don’t need more information right now. I will consider attending caregiver education eventually.”*

In the group with a diagnosis, everyone chose to be open about the diagnosis and the challenges, and they experienced support and understanding from their surroundings. They reported well-functioning family ties with help and support from children, neighbours, and siblings. Molly was very grateful to her children who visits every day: *“They are kind and always there for us.”* She was grateful for what she described as a happy marriage for 60 years and didn’t miss anything: *“I have what I need. We are doing well. I am doing well.”*

Among caregivers to family members without a dementia diagnosis, there was a tendency to emphasize that everything was fine and that no needs were unmet: *“I am very happy with how they (the parents) live today. They have freedom and enjoy themselves out there”* (Eric). Emma was also eager to emphasize that she and her husband were doing well and that there was nothing she wanted to change. She also mentioned the value of a supportive community: *“We live in an apartment building with others of the same age. We support each other.”*

#### Impacts on the caregivers’ quality of life

Although caregivers generally reported satisfaction with the support and services they received, many felt increasingly isolated. Martin mentioned a restricted social life due to his wife’s fear of going out: *“We stay home a lot because she is afraid to go out.”* Similarly, Mia described the monotony and difficulty in motivating her husband for social activities: *“I want to attend concerts and enjoy music*,* but he is reluctant*,* and I often have to persuade him to join family dinners.”* She also noted a decline in their social circle: *“We have less contact with some friends than we used to.”*

Mia also expressed feeling increasingly restricted in their social engagements and ability to leave home: *“I could participate in activities*,* but when he doesn’t want to join*,* I find it hard to leave him.”* Meanwhile, Martin highlighted the limitations imposed by his wife’s inability to stay home alone: *“It’s difficult for me to get away. She cannot be home alone.”* In contrast, Maria discussed her ability to travel without major concerns, although she noted practical challenges related to her husband’s independence and need for support in making warm food for dinner without her being there.

When it came to future prospects, caregivers of family members without a diagnosis expressed a lack of concern about the future: *“I’m not thinking about the future. We’re truly happy”* (Emma). Eric was the exception, expressing worry about potential deterioration in his father’s cognition. In contrast, caregivers of those with a dementia diagnosis were cautious yet realistic about what the future might bring. Mia acknowledged the possibility of eventually being alone but expressed a commitment to staying with her husband for as long as possible. She also voiced concerns about the possibility of her husband needing nursing home care: *“I’m very afraid that he will end up in a nursing home and be lost to us*,* not recognizing us anymore.”* Maria said: *“We know what needs may arise*,* but he’s old now and won’t live with this disease for another 20 years. Other issues will come up too.”* While Martin concluded: *“I think we have reached the final round of life’s waltz.”*

## Discussion

This study aims to explore the implications of a family member with cognitive impairment receiving a dementia diagnosis or not from the perspective of their next of kin.

The findings show that caregivers who have received a dementia diagnosis for their family members are more proactive in seeking help and services, are better informed about available resources, and are more concerned about future challenges. On the other hand, caregivers without a diagnosis are more inclined to handle the situation on their own, have less access to information and services, and generally express less concern about future problems.

### Diagnosis and communication

Despite all caregivers noting cognitive decline in their family members, only five considered that further investigation into cognitive function was necessary. Additionally, one caregiver attributed the cognitive decline to either incorrect medication or improper management of type 2 diabetes. This underscores the importance of healthcare providers assessing cognitive function within the broader context of an individual’s overall health [26]. The study suggests a potential gap in dementia care, indicating that further information about the benefits and process of obtaining a diagnosis might be beneficial [27].

Interestingly, two caregivers reported that diagnosis and treatments were initiated without explicit discussions or confirmations from healthcare providers. In both cases, dementia medication was given without a formal diagnosis being communicated or perceived. This finding suggests a potential system-level issue in the management and information flow within the healthcare system, as also suggested by Burgdorf et al. [28]. These findings underscore the importance of clear communication and comprehensive caregiver education by healthcare professionals, not only regarding the diagnosis but also with respect to treatment options and ongoing management. When a formal dementia diagnosis is established, healthcare personnel should ensure that information regarding the diagnosis and subsequent follow-up is understood by both the affected person and their family caregivers. This finding aligns with the results of previous studies [29, 30].

### Impact of diagnosis on caregiver service utilization

Our findings indicate that the use of services depends on the extent of available assistance from family and close relationships, as well as the need for independence and self-efficacy. Other studies have shown that self-efficacy is an effective coping strategy that provides confidence and independence for informal caregivers [31, 32].

Awareness of services was significantly greater among family caregivers of individuals diagnosed with dementia than among those of individuals without a dementia diagnosis. Caregivers navigated a delicate balance between fostering independence and self-management for themselves and their relative in addition to providing necessary care. Additionally, there was a significant discrepancy in the awareness and utilization of services between the two groups of caregivers. Those caring for formally diagnosed individuals showed greater engagement with a broad array of services, suggesting that a diagnosis serves not only as a clinical marker but also as a key determinant in accessing crucial support. Formal diagnoses often act as catalysts, prompting caregivers to seek out educational resources, support groups, and medical interventions that can improve the quality of care [33, 34]. The diagnosis effectively legitimizes the caregivers’ concerns and opens doors to specialized resources that might otherwise remain inaccessible or unknown.

In contrast, caregivers without a formal diagnosis tended to adopt a more self-reliant approach. This group was less likely to seek external assistance, perhaps due to a lack of recognition of the severity or nature of the cognitive impairment. The absence of a formal diagnosis may contribute to a sense of ambiguity and uncertainty, leading caregivers to rely on their own intuition and resources rather than seeking professional help. This self-reliance can also result in missed opportunities for beneficial interventions and support.

### Information and service accessibility

According to other studies [30, 35], caregivers with a formal diagnosis are generally better informed about available resources, including medical treatments, financial aid, and community services. This enhanced access to information is critical in navigating the complex and often fragmented landscape of dementia care. Well-informed caregivers are better equipped to advocate for their family members, make informed decisions, and manage the multifaceted challenges associated with dementia [30].

Our findings indicate that caregivers without a formal diagnosis were generally less informed about where to seek help if symptoms were to increase and unfamiliar with available services and information about cognitive impairment and dementia. These findings underscore the importance of a formal diagnosis to access adequate information, education, treatment, and support, not only for the sake of the patient but also for empowering caregivers.

### Caregiver stress: the impact of dementia diagnosis

Family caregivers play a critical role in the lives of individuals with dementia, often providing the majority of care and support [36, 37]. Their experiences are shaped by the presence or absence of a formal diagnosis [34, 38]. In our study, caregivers of diagnosed individuals tended to experience a sense of validation and a clearer pathway to access resources and support. However, these caregivers also faced the emotional burden of the diagnosis and the demands of managing the disease progression. Studies have shown that caregivers often experience high levels of stress, anxiety, and depression, which can adversely affect their health and well-being [16, 29, 31]. Caregivers of diagnosed individuals may experience a mix of relief and burden [28, 30]. Relief arises from understanding the condition and accessing support, whereas the burden stems from the demands of caregiving and the emotional impact of the diagnosis. Conversely, without a formal diagnosis, accessing the necessary support and information can be challenging, leading to increased stress and a sense of helplessness. These caregivers may struggle to understand the cognitive impairments and behavioural changes in family members, making it difficult to provide appropriate care and support.

### Caregiver concerns and timely dementia diagnosis

Concerns about the future also varied between the two groups of caregivers. The caregivers of family members with a dementia diagnosis expressed greater concern about future challenges. Caregivers without a dementia diagnosis chose not to worry about the future but preferred to live each day as it came. Other studies have shown similar results [16], including that people with dementia feel apprehensive about the future [24]. In our study, two out of three caregivers of persons without a dementia diagnosis reported being content with their life and not wishing to change anything. Timely diagnosis means recognizing dementia once symptoms are noticeable but before significant decline. This can be related to discission about timely versus early dementia diagnosis, whereas timely diagnosis holds key advantages for pharmacological treatment as well as nonpharmacological interventions and services, all of which can significantly enhance outcomes for patients and their families. A timely diagnosis allows caregivers to educate themselves about the condition, seek out support networks, and prepare for the increasing demands of caregiving. Timely diagnosis is beneficial because it allows those with dementia and their families to make decisions about the future while they are still capable of doing so [39, 40]. However, it is important to consider that the concept of timeliness can be subjective, as withholding a diagnosis until it seems timely may not always be accurate or appropriate. Further research is needed to explore these contrasting perspectives and to understand the implications of different approaches to diagnosis, ensuring they truly support the rights and wellbeing of individuals with dementia and their families.

Even so, ignorance or avoidance of a dementia diagnosis might be an adequate coping strategy for maintaining one’s identity and quality of life [30, 32].

## Strengths and limitations

The aim of our study was to explore the subjective experiences of the participants in terms of receiving a diagnosis or not, from the perspective of family caregivers. Interesting contrasts in the sharing of experiences between the two groups emerged from the data. Importantly, the information regarding whether caregivers were providing care for someone with or without a dementia diagnosis was based on self-reports. To ensure trustworthiness and reflexivity, two coauthors (GK and AMMR) were involved in the analysis and interpretation of the data.

The sample size in this study was small, consisting of only eight participants. In conducting this research, we adhered to the concept of ‘information power’ [41], which prioritizes the relevance and richness of data over sheer sample size. This approach suggests that the more relevant and specific the information, the fewer participants are needed. Despite practical constraints limiting our sample to eight interviews, the data they provided was highly relevant and aligned with our research objectives, offering deep insights into the variations in caregiver experiences.

The variation in age and caregiver role (spouse or son) adds complexity to generalizing the perspective of caregivers. The group of individuals with dementia had lower scores and an older average age compared to those without a diagnosis, which makes it challenging to directly compare the experiences of the caregivers.

## Conclusion

Understanding the experiences of family caregivers is crucial for improving dementia care and support. This study aims to provide a comprehensive exploration of the implications of receiving a dementia diagnosis from the caregivers’ perspective. The findings highlight the importance of transparent communication between healthcare professionals, patients, and family caregivers to facilitate better understanding, management, and support for those with dementia. Furthermore, understanding the nuanced impacts of early versus timely diagnosis can help shape more effective support systems and policies, ensuring that caregivers and patients alike can navigate the complexities of dementia with greater confidence and preparedness and enhancing the quality of life for both individuals with dementia and their caregivers.

## Data Availability

Information about age, gender, phone number, and place of residence is stored with an ID number (not name) in a separate file on the secured research server of the National Centre for Aging and Health. The data cannot be shared openly to protect study participants’ privacy, which aligns with the content in their written consent. To request access to the study data, please contact the corresponding author.
